# Book Reviews

**Published:** 1902-01

**Authors:** 


### BOOK REVIEWS.

THE PRACTICAL MEDICINE SERIES OF YEAR BOOKS. Comprising ten volumes of the year's progress in medicine and surgery. Issued monthly. Under the general editorial charge of Gustavus P. Head, M.D., Chicago. Vol. 1. General medicine. Edited by Frank Billings, U. S., M.D., with the collaboration of S. C. Stanton, M.D., October, 1901. Price of the series $7.50, of this volume $1.50. The Year Book Publishers, 40 Dearborn street, Chicago, Ill.

This series is designed as an inexpensive method of keeping the medical reader informed of the progress of medicine in its several directions. The first volume is devoted to general medicine and contains an excellent digest of new views and facts which have been brought out recently in this department. It includes diseases of the respiratory and circulatory organs, infectious and constitutional diseases, and a chapter on miscellaneous diseases. The series is recommended to those who desire late information on any topic in medicine.

---

PROGRESSIVE MEDICINE, Vol. IV., December, 1901. A Quarterly Digest of Advances, Discoveries and Improvements in the Medical and Surgical Sciences. Edited by Hobart Amory Hare, M.D., Professor of Therapeutics and Materia Medica in the Jefferson Medical College of Philadelphia. Octavo, handsomely bound in cloth, 398 pages. Per annum, in four clothbound volumes, $10.00. Lea Brothers & Co., Philadelphia and New York.

The present volume deals with diseases of the Digestive tract and allied organs, the Liver, Pancreas and Veritoreum, Genito-Urinary Diseases, Anesthetics, Fractures, Dislocations, Amputations, Surgery of the Extremities and Orthopedics, Diseases of the Kidney (non-surgical), Physiology, Hygiene, Practical Therapeutic Referendum. The contributors are Max Einbom, J. C. Bloodgood, J. R. Bradford, Albert P. Brubaker, Henry B. Baker,

E. Quin Thornton. The volume is well illustrated and printed, and is fully up to the standard created by previous issues.

---

OUTLINES OF PHYSIOLOGY. By Edward Grove Jones, M.D., Atlanta, Ga., Lecturer on Physical Diagnosis in the Atlanta College of Physicians and Surgeons, and professor of Physiology in the Dental department of the same. P. Blackiston's Son & Co., Philadelphia, 1901.

Dr. Jones is to be congratlated upon the production of a book that will prove of much benefit to the student launching out upon the difficult science of physiology. The work is designed to simplify the subject of physiology and to present its essential facts in a brief and concise manner. Theoretical discussion and argumentation is eliminated as leading to confusion and deflection from the main purpose. The little book will be welcomed by medical students everywhere, and it is to be cordially recommended.

---

LIBERTINISM AND MARRIAGE. By Dr. Louis Jullien. Translated by R. B. Douglas. 12mo, pp. 169! Philadelphia: F. A. Davis Company. 1901. Price, $1.00 net, delivered.

This translation from the noted French genito-urinary surgeon, Jullien, deals with the question of gonorrhoea and marriage under varying circumstances. After a chapter on professional discretion, the proper course of conduct of both physician and patient, whether male or female, is indicated before the betrothal of the latter, after betrothal and after marriage. The book contains much sage counsel and advice, and is written from an abundance of experience on the part of the author. The translation is somewhat too literal for ease of expression.

---

GRAY'S ANATOMY—DESCRIPTIVE AND SURGICAL. By Henry Gray, F. R. S. Edited by T. Pickering Pick, F. R. C. S. Philadelphia and New York. Lea Bros. & Co., 1901.

Gray's Anatomy is one of the landmarks of medicine. It had modest beginnings, but edition after edition have enlarged and perfected it until its name is almost synonymous with the science of anatomy. The present edition contains, besides those familiar for years, many new and fine illustrations, and some amplification of text and rearrangement.

---

PRACTICAL MEDICINE SERIES. Vol. 2. November, 1901. General Surgery. Edited by John B. Murphy, M.D., Chicago. Price $2.00. The Year Book Publishers, 40 Dearborn street, Chicago.

The second volume of this series is devoted to the subject of general surgery, and is under the editorship of Dr. John B. Murphy. It contains a summary of recent progress in surgery and fairly covers the whole field. There are a few illustrations. The modest price of these books and the usefulness of their character commend them to the practitioner, and a perusal warrants an endorsement.

---

### *SELECTIONS AND ABSTRACTS.*

---

### HYDROPHOBIA, WITH REPORT OF THREE CASES*

Of the three cases which I wish to report to-night, two occurred under my own observation in 1899, and the third is of recent occurrence in the practice of Dr. Thomas Hubbard, of Toledo, who kindly allows me to report it with my own.

The first patient, Mr. L., 71 years of age, was seen at the request of Drs. J. F. and W. S. Hobson, on May 30, 1899. Until the development of this difficulty he was in good health. During the very cold weather early in February he went to the barn to milk, and was bitten on the hand by a large tomcat, which sprang upon him without obvious reason. He had been in the habit of petting the cat, which seemed to be fond of him. He allowed about one-half hour to elapse, and the wound was then cauterized and treated with an antiseptic dressing. The cat had been observed to be sick

* Read by Henry S. Upson, M.D., Professor of Diseases of the Nervous System in the Western Reserve Medical School, Cleveland, before the Cleveland Medical Society, October 11, 1901.

